# Efficacy and Safety of Regional Citrate Anticoagulation in Neurocritical Care Patients With Chronic Severe Hypernatremia Undergoing Continuous Renal Replacement Therapy: A Single-Center Retrospective Study

**DOI:** 10.14740/jocmr6321

**Published:** 2026-01-16

**Authors:** Gui Zhen Zhu, Xiao Min Gong, Yang Lu, Xu Ma, Guo Sheng Yan, Jing Yi Wan, Hong Tao Zhang

**Affiliations:** a Department of Nephrology, People’s Hospital of Zhengzhou University, Zhengzhou, China; b Blood Purification Center, Henan Provincial People’s Hospital, Zhengzhou, China; c Department of Nephrology, Fuwai Central China Cardiovascular Hospital, Zhengzhou, China; d These authors contributed equally to this work.

**Keywords:** Continuous renal replacement therapy, Chronic severe hypernatremia, Neurocritical care patients, Regional citrate anticoagulation, High risk of bleeding

## Abstract

**Background:**

Hypernatremia is a common complication among neurocritical care patients. This study aimed to investigate the effectiveness and safety of regional citrate anticoagulation (RCA) vs. no anticoagulation (NA) in neurocritical patients receiving continuous renal replacement therapy (CRRT) who also had chronic severe hypernatremia and an elevated risk of bleeding.

**Methods:**

From March 2020 to August 2024, electronic medical records of neuro-critically ill patients who underwent CRRT for chronic severe hypernatremia with elevated risk of bleeding at Henan Provincial People’s Hospital’s neurocritical intensive care unit (ICU) were retrospectively analyzed. Patients were divided into RCA (n = 70) and NA (n = 28) groups. The key effectiveness objective was the mean serum sodium correction, while the primary safety event was the occurrence of common anticoagulant adverse events. Original cohorts were matched using propensity score matching (PSM) between two groups (n = 21). Risk factors impacting the initial filter lifespan were analyzed using Cox proportional risk regression model.

**Results:**

Both groups achieved similar sodium correction rates (0.5 ± 0.1 mmol/L/h). The RCA group had a lower incidence of both hemorrhagic (6/70 (8.6%) and 8/28 (28.6%), P = 0.021) and filter coagulation (0/70 (0%) and 17/28 (60.7%), P < 0.001) adverse events. After performing Kaplan-Meier curve and multivariable Cox regression, RCA was identified as an independent protective factor for first filter lifespan (hazard ratio (HR) = 0.09, 95% confidence interval (CI), 0.05–0.18).

**Conclusion:**

RCA is safer and equally effective as NA for CRRT in neurocritical patients with chronic severe hypernatremia, reducing bleeding and filter clotting risks. While our retrospective study suggests that RCA is a safe and effective strategy in this population, the findings require validation in a large-scale, randomized controlled trial to establish conclusive evidence.

## Introduction

Hypernatremia is a frequent complication in patients with severe neurologic disorders, occurring in nearly 50% of neurocritical care cases [1, 2]. Among traumatic brain injury (TBI) patients, its incidence reaches 36.9%, often due to thirst center dysfunction, sympathetic stress, reduced natriuretic peptides, and osmotic therapy [3, 4]. Severe hypernatremia (> 160 mmol/L) independently predicts mortality, with rates as high as 86.8% [1, 5].

Rapid correction of chronic hypernatremia is contraindicated due to risks of cerebral edema, herniation, and irreversible neurologic damage [6]. Current guidelines recommend: a correction rate ≤ 0.5 mmol/L/h and a maximum daily reduction of ≤ 12 mmol/L [7]. In critically ill neurologic patients with severe hypernatremia, continuous renal replacement therapy (CRRT) is a significant therapeutic option since it can progressively, controllably, and continuously lower blood salt content [1]. The lifespan of the filter has a direct bearing on how adequate CRRT is. Extracorporeal circuit coagulation is the main cause of circuit clotting, which frequently occurs during CRRT [8]. However, circuit clotting is a major CRRT complication, which can lead to increased blood loss, nursing workload, shorter treatment times, lower treatment efficacy, and higher hospitalization expenses [9, 10]. Neurologic patients often face elevated bleeding risks (e.g., coagulopathy, recent surgery/trauma), leading some clinicians to avoid anticoagulation despite its benefits for filter longevity. Regional citrate anticoagulation (RCA) has risks and controversies in patients with hypernatremia. Per Kidney Disease: Improving Global Outcomes (KDIGO) guidelines, RCA is preferred for bleeding-prone CRRT patients without citrate contraindications [11]. However, 4% trisodium citrate (TSC) carries a high sodium load (408 mmol/L), potentially exacerbating hypernatremia with prolonged use. Thus, RCA remains debated in severe hypernatremia, with some guidelines labeling it a relative contraindication [12]. No research compared the safety and efficacy of RCA vs. no anticoagulation (NA) therapy for in critically ill neurologic patients with chronic severe hypernatremia.

## Materials and Methods

### Study design and population

Medical records of patients aged 18 and older who were hospitalized to Henan Provincial People’s Hospital’s neurosurgery ICU between March 2020 to August 2024 and received CRRT for persistent severe hypernatremia with bleeding tendency were reviewed retrospectively. Inclusion criteria were those who received CRRT for persistent chronic severe hypernatremia, defined as a serum Na^+^ ≥ 160 mmol/L documented for a duration of > 48 h or with an unknown time of onset, consistent with the pathophysiological state of chronicity and associated brain adaptation [4]; who had bleeding risk (platelets < 10 × 10^9^/L, activated partial thromboplastin time (aPTT) > 60 s, international normalized ratio (INR) > 2.0, active bleeding, recent surgery (≤ 7 days), or intracranial hemorrhage (≤ 3 months)) [11]. Exclusion criteria were missing critical data (e.g., blood gases), concurrent systemic anticoagulation. The patient’s financial situation will influence the attending physician’s choice of anticoagulation modality. To address potential confounding factors arising from this non-randomized allocation, we performed 1:1 propensity score matching (PSM) to create balanced groups for comparison. The clinician determined the patient’s anticoagulation modality, and patients were separated into RCA group and NA group. This retrospective study was approved by the Ethics Committee of People’s Hospital of Zhengzhou University (approval no. 2023-0145), and performed in accordance with the Declaration of Helsinki and waived the need for informed consent because of the retrospective study design.

### CRRT prescription

All patients received temporary vascular access via a double-lumen central venous catheter (13.5F, Bard, USA) inserted into the femoral vein. Continuous venovenous hemodiafiltration (CVVHDF) was performed using the Prisma Flex machine (Gambro, Sweden). For filter selection, M150 (AN69 membrane, area 1.25 m^2^) was adopted. Post-dilution replacement fluid rates were 1,000 mL/h, dialysis fluid rates were 1,000 mL/h, and blood flow rates were regulated between 150 and 200 mL/min. A prescription dose of 25 mL/kg/h was first given. Commercialized replacement fluid (Chengdu Qingshan Likang Pharmaceutical Co., Ltd.) was used. Hemofiltration basic solution (4,000 mL) contained the following: Na^+^: 113 mmol/L, Cl^−^: 118 mmol/L, Ca^2+^: 1.60 mmol/L, Mg^2+^: 0.979 mmol/L, glucose: 10.6 mmol/L.

For patients in the RCA group, the anticoagulant citrate solution (200 mL: 8.0 g, produced by Sichuan Nange Biological Technology Co., Ltd.) was infused through the pre-buffer pump (PBP) of the CRRT machine from the arterial end. Calcium gluconate was added to the post-dilution fluid to replenish the lost calcium ions. The 5% sodium bicarbonate (NaHCO_3_) solution was continuously infused via the central venous catheter (CVC). The infusion rate of sodium bicarbonate (mL/h) was calculated according to the following formula: (Dialysis fluid rate + Replacement fluid rate) × 62.5 − 1/2 Citrate sodium rate. Blood gas analysis was performed every 4 h, and prescription parameters were adjusted immediately according to the results. In the RCA group, the post-filter calcium was maintained at 0.25–0.40 mmol/L, and RCA dose was dynamically adjusted based on serum calcium levels to maintain free calcium within 0.9–1.2 mmol/L.

For patients in the NA group, the extracorporeal circuit was established without the use of any anticoagulant. The circuit was primed exclusively with saline. To mitigate the risk of circuit clotting inherent to this approach, a standardized protocol was followed. Regular saline flushing: the circuit was intermittently flushed with 100–150 mL of saline every 4–6 h or whenever there was a trend of increasing transmembrane pressure. Circuit management: circuit patency was vigilantly monitored through continuous pressure monitoring. However, it was replaced immediately if signs of significant clotting (e.g., a rapid rise in transmembrane pressure > 250 mm Hg, visible clot formation in the bubble trap or venous chamber) were observed. All other CRRT parameters, including the use of post-dilution CVVHDF mode, blood flow rate, dialysate, and replacement fluid composition and rates, were identical to those described for the RCA group, ensuring that the anticoagulation strategy was the sole major variable.

Bleeding, extracorporeal circuit coagulation, and filter pressure were all continually tracked, with a scheduled replacement of the filter every 72 h. The initial sodium concentration of the replacement fluid was adjusted to be 8 mmol/L lower than the serum sodium level by adding 10% NaCl to the replacement fluid, followed by gradual reduction every 4 h.

### Data collection and definitions

Our hospital database was retrospectively searched for demographic data such as age, gender, hypertension, and diabetes mellitus at the time of diagnosis of chronic severe hypernatremia, as well as clinical data such as whether mechanical ventilation was used, underlying disease, classification of neurological disease, whether cranio-cerebral surgery was performed, use of osmotic diuretics, high-dose mannitol (> 100 g/d), ICU length of stay, and total hospitalization costs. Acute kidney injury (AKI) was diagnosed according the KDIGO criteria, characterized by an increase in serum creatinine (SCr) 0.3 mg/dL within 48 h and a decrease in urine volume < 0.5 mL/kg every hour for 6 h. On the day of CRRT initiation, the disease severity was assessed using the Acute Physiology and Chronic Health Evaluation II (APACHE II) and Glasgow Coma Scale (GCS) grading methods, and urine output was documented. Laboratory data and CRRT-related date were gathered pre-and post-CRRT, including duration of CRRT (hours), lifespan of the first filters (hours), number of filters used, and the occurrence of adverse events.

### Study outcomes

The main effectiveness outcomes were the serum sodium concentration correction rate. Change in serum sodium concentration (mmol/L)/treatment time (hours) is the formula used to calculate the serum sodium concentration correction rate. The filter lifespan was defined as the time from the onset of extracorporeal blood circulation to the termination of the circulation owing to circuit coagulation.

The primary safety outcome was the occurrence of anticoagulation-related adverse events during CRRT, such as bleeding (from drainage tubes or progressive hemoglobin (Hb) decrease), frequent filter clotting changes (< 24 h), acid-base balance disorders (metabolic alkalosis, metabolic acidosis), hypocalcemia (serum calcium level < 0.7 mmol/L), and citrate accumulation (criteria: total calcium/ionized calcium (TCa/iCa^2+^) ratio > 2.5 and clinically significant hypocalcemia that does not improve with calcium administration).

### Statistical analysis

We performed all statistical analyses with R version 4.2.1 (R package for Statistical Computing). Categorical variables were presented as the number (percentage), and continuous variables were presented as mean (standard deviation). Normally distributed continuous variables were compared using the *t*-test; otherwise, the non-parametric Kruskal-Wallis H test was applied. The Chi-square test was used to evaluate categorical variables. Multiple interpolation was employed to deal with missing data.

PSM was performed using the nearest neighbor method with a 1:1 ratio and a caliper width of 0.1 to balance baseline characteristics between the RCA and NA groups. The propensity score was estimated using a logistic regression model that included the following covariates: age, sex, APACHE II score, GCS score, mechanical ventilation, neurological disease category, history of craniocerebral surgery, hypertension, diabetes, use of osmotic diuretics, AKI, urine output, serum sodium, total calcium, serum creatinine, serum urea, Hb, platelet count, prothrombin time, aPTT, serum albumin (ALB), and total bilirubin. The final sample size after PSM (n = 21 per group) was determined by the number of eligible patients available for matching from the original cohort. While this sample size is modest and may limit the statistical power for detecting differences in secondary outcomes, it is consistent with other single-center retrospective studies focusing on this specific and critically ill patient population. The primary goal of PSM in this context was not to achieve a predetermined sample size for power calculation, but rather to minimize selection bias and create comparable groups for a more robust comparison of the primary outcomes (filter lifespan and bleeding events) between the two anticoagulation strategies.

We estimated the lifespan of the first filter using the Kaplan-Meier curve. We utilized multivariate Cox proportional risk regression models to identify independent factors influencing the lifespan of the first filter, which were reported as hazard ratios (HRs) and 95% confidence intervals (CIs). The univariate Cox regression analysis included all baseline variables, which were checked to ensure that the proportional risk assumption was met prior to doing the Cox regression analyses. We chose factors with significant impacts (P *<* 0.05) in univariate analysis for multivariate Cox proportional hazards regression. In multivariate Cox proportional hazards regression, we determined the degree of multicollinearity between variables and evaluated correlation analyses between covariates using variance inflation factor (VIF) studies. A P-value of less than 0.05 was judged statistically significant.

## Results

### Characteristics of the study cohort

**Figure 1:**
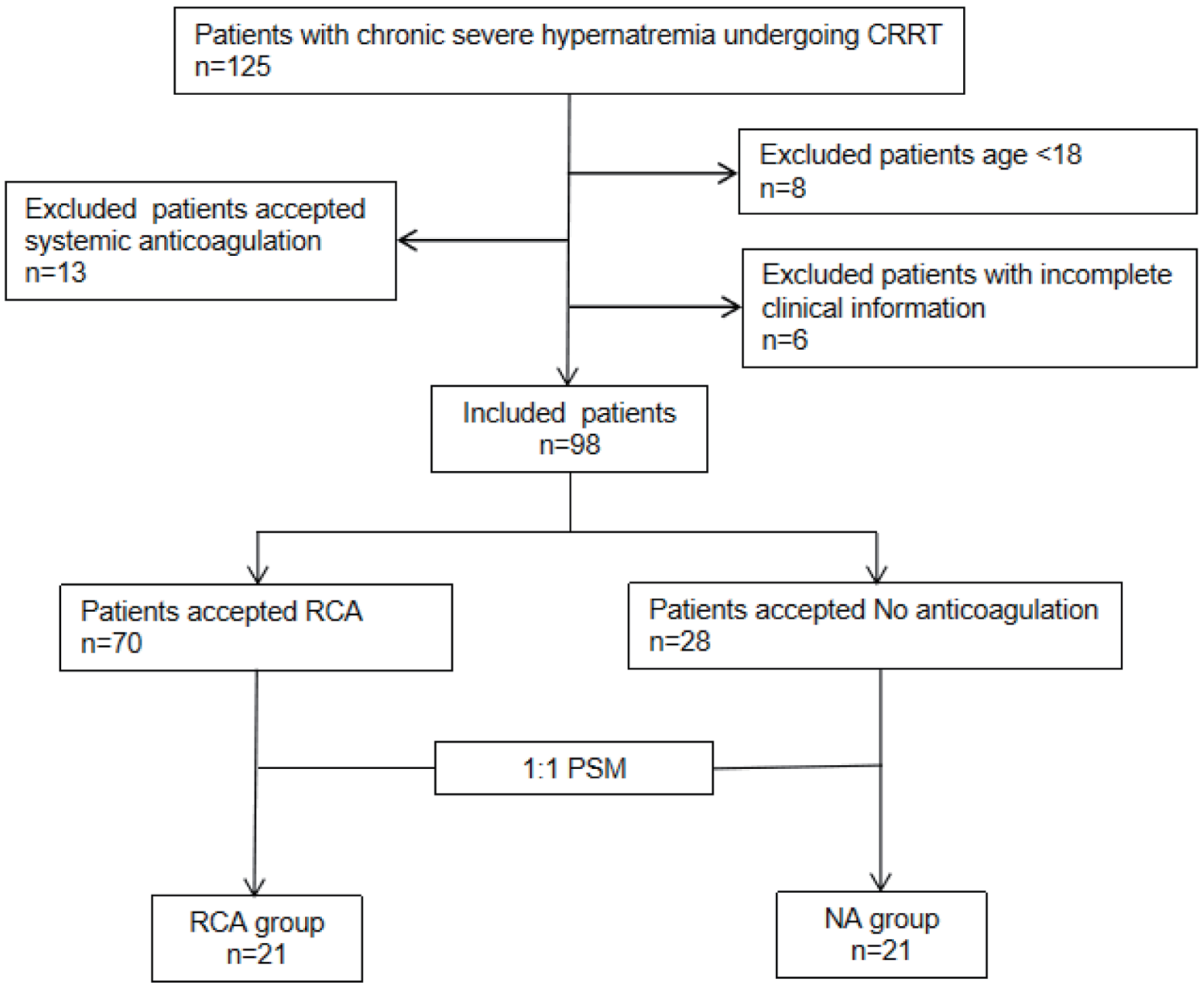
Flow chart of enrollment. Included patients (n = 98) were divided into two groups: regional citrate anticoagulation (RCA) groups (n = 70) and no anticoagulation (NA) groups (n = 28). A 1:1 propensity score matching (PSM) was performed to create two balanced groups: “NA group” and “RCA group,” each with n = 21 patients.

From March 2020 to August 2024, 125 neurocritical care patients at high bleeding risk with chronic severe hypernatremia undergoing CRRT were screened (Fig. 1). Twenty-seven patients were excluded due to age < 18 years (n = 8), use of systemic anticoagulation (n = 13), and incomplete clinical data (n = 6). Ninety-eight patients were enrolled: 70 in the RCA group and 28 in the NA group. AKI incidence (P = 0.048) and creatinine levels (P < 0.001) differed significantly between groups. Forty-two patients were successfully matched using PSM. The baseline characteristics of the patients were compared according to pre- and post-matching (Table 1).

**Table 1:** Baseline Characteristics of Patients Before and After PSM

Variables	Before PSM				After PSM			
	Total (n = 98)	RCA (n = 70)	NA (n = 28)	P	Total (n = 42)	RCA (n = 21)	NA (n = 21)	P
Male	69 (70.4)	49 (70)	20 (71.4)	0.889	33 (78.6)	16 (76.2)	17 (81)	> 0.99
Age, years	53.0 ± 14.5	52.5 ± 13.0	54.2 ± 17.9	0.603	53.5 ± 16.7	51.5 ± 14.6	55.4 ± 18.8	0.461
APACHE II score	28.3 ± 4.5	27.9 ± 4.5	29.2 ± 4.5	0.213	28.4 ± 3.2	28.8 ± 3.0	28.0 ± 3.4	0.448
GCS score	3.0 ± 1.8	3.2 ± 2.0	2.6 ± 1.2	0.146	2.8 ± 1.1	2.7 ± 1.0	2.8 ± 1.3	0.791
Mechanical ventilation	86 (87.8)	62 (88.6)	24 (85.7)	0.738	40 (95.2)	20 (95.2)	20 (95.2)	> 0.99
Neurologic disease				0.429				0.156
Traumatic brain injury	35 (35.7)	27 (38.6)	8 (28.6)		15 (35.7)	7 (33.3)	8 (38.1)	
Intracerebral hemorrhage	39 (39.8)	25 (35.7)	14 (50)		21 (50.0)	13 (61.9)	8 (38.1)	
Subarachnoid hemorrhage	4 (4.1)	4 (5.7)	0 (0)		0 (0)	0 (0)	0 (0)	
Other categories	20 (20.4)	14 (20)	6 (21.4)		6 (14.3)	1 (4.8)	5 (23.8)	
Post craniocerebral operation	74 (75.5)	55 (78.6)	19 (67.9)	0.265	30 (71.4)	15 (71.4)	15 (71.4)	> 0.99
Comorbidity								
Hypertension	50 (51.0)	34 (48.6)	16 (57.1)	0.443	24 (57.1)	11 (52.4)	13 (61.9)	0.533
Diabetes	23 (23.5)	14 (20)	9 (32.1)	0.2	11 (26.2)	4 (19)	7 (33.3)	0.292
Use of osmotic diuretics	83 (84.7)	61 (87.1)	22 (78.6)	0.353	36 (85.7)	18 (85.7)	18 (85.7)	> 0.99
AKI	70 (71.4)	46 (65.7)	24 (85.7)	0.048	33 (78.6)	16 (76.2)	17 (81)	> 0.99
AKI stage				0.003				0.616
AKI stage 1	39 (39.8)	30 (42.9)	9 (32.1)		16 (38.1)	8 (38.1)	8 (38.1)	
AKI stage 2	20 (20.4)	13 (18.6)	7 (25.0)		10 (23.8)	6 (28.6)	4 (19.0)	
AKI stage 3	11 (11.2)	3 (4.3)	8 (28.6)		7 (16.7)	2 (9.5)	5 (23.8)	
Urine volume, mL	3,415.1 ± 1,776.0	3,514.0 ± 1,715.4	3,167.8 ± 1,929.5	0.386	3,502.2 ± 1,684.6	3,597.8 ± 1,593.8	3,406.6 ± 1,805.1	0.718
Serum sodium, mmol/L	171.7 ± 8.9	172.3 ± 9.4	170.4 ± 7.7	0.349	171.2 ± 8.4	170.2 ± 9.3	172.1 ± 7.6	0.472
Total calcium, mmol/L,	2.3 ± 0.2	2.3 ± 0.2	2.3 ± 0.2	0.304	2.3 ± 0.2	2.4 ± 0.3	2.3 ± 0.2	0.523
Serum creatinine, µmol/L,	176.3 ± 127.2	147.8 ± 95.7	247.8 ± 165.2	< 0.001	205.3 ± 132.4	195.2 ± 113.1	215.3 ± 151.5	0.629
Serum urea, mmol/L	17.4 ± 11.3	16.5 ± 11.3	19.8 ± 11.1	0.191	20.3 ± 11.2	20.5 ± 9.8	20.1 ± 12.7	0.894
Hb, g/L	107.1 ± 23.5	104.8 ± 24.0	112.6 ± 21.7	0.138	111.4 ± 23.3	112.2 ± 25.6	110.6 ± 21.4	0.83
PLT count, × 10^9^/L	139.7 ± 80.4	144.8 ± 79.1	126.9 ± 83.6	0.321	142.0 ± 80.5	146.6 ± 71.3	137.3 ± 90.4	0.715
PT (s)	14.9 ± 3.2	15.0 ± 3.5	14.8 ± 2.4	0.768	14.6 ± 1.7	14.8 ± 1.5	14.4 ± 1.9	0.484
aPTT (s)	30.6 ± 8.2	30.5 ± 8.7	30.6 ± 7.1	0.976	30.3 ± 6.0	31.2 ± 6.0	29.5 ± 5.9	0.369
Serum ALB, g/L	34.6 ± 5.9	34.0 ± 6.1	36.0 ± 5.1	0.133	36.5 ± 6.2	36.1 ± 7.3	37.0 ± 5.1	0.674
Serum total bilirubin, mmol/L	16.6 ± 13.3	15.8 ± 13.6	18.6 ± 12.5	0.357	16.4 ± 10.1	15.8 ± 10.5	16.9 ± 10.0	0.724

ALB: albumin; AKI: acute kidney injury; APACHE II: Acute Physiology and Chronic Health Evaluation II; aPTT: activated partial thromboplastin time; GCS: Glasgow Coma Scale; Hb: hemoglobin; NA: no anticoagulation; PLT: platelet; PSM: propensity score matching; PT: prothrombin time; RCA: regional citrate anticoagulation.

### Comparative effectiveness and safety of RCA vs. NA

Before CRRT, serum sodium concentrations were 172.3 ± 9.4 and 170.4 ± 7.7 mmol/L in the RCA and NA groups, respectively (P = 0.349). After CRRT, serum sodium levels were 147.9 ± 6.8 and 150.5 ± 7.2 mmol/L in the RCA and NA groups, respectively (P = 0.094). The average length of CRRT in the RCA group was 52.6 ± 22.2 h, while in the NA group, it was 40.8 ± 21.2 h (P = 0.017). The rate of serum sodium correction was 0.5 ± 0.1 mmol/L/h in the RCA group and 0.5 ± 0.1 mmol/L/h in the NA group, with no significant difference between the two groups (P = 0.587). During CRRT, six patients in the RCA group and eight patients in the NA group experienced bleeding (6/70 (8.6%) and 8/28 (28.6%), P = 0.021). In the NA group, as many as 17 patients suffered from frequent filter coagulation adverse events (17/28 (60.7%), P < 0.001), but no such events were observed in the RCA group (0/70 (0%), P < 0.001). No significant differences in metabolic acidosis, alkalosis, or hypocalcemia (P > 0.05) were observed. Citrate accumulation was observed in six patients in the RCA group (6/70, 8.6%), which all were corrected in subsequent treatment. After PSM, the risk of bleeding event was significantly lower in patients treated with RCA than in patients treated with NA (0/21 (0%) and 5/21 (23.8%), P = 0.048), as was the occurrence of frequent filter clotting (0/21 (0%) and 12/21 (57.1%), P < 0.001) (Table 2).

**Table 2:** Outcomes of Serum Sodium Reduction Rate and Adverse Events

Variables	Before PSM				After PSM			
	Total (n = 98)	RCA (n = 70)	NA (n = 28)	P value	Total (n = 42)	RCA (n = 21)	NA (n = 21)	P value
Post CRRT serum sodium, mmol/L	148.6 ± 7.0	147.9 ± 6.8	150.5 ± 7.2	0.094	148.8 ± 7.1	146.8 ± 6.1	150.8 ± 7.6	0.068
Mean hypernatremia correction rates, mmol/L/h	0.5 ± 0.1	0.5 ± 0.1	0.5 ± 0.1	0.587	0.5 ± 0.1	0.5 ± 0.2	0.5 ± 0.1	0.613
CRRT duration, h	49.2 ± 22.5	52.6 ± 22.2	40.8 ± 21.2	0.017	45.9 ± 19.7	49.2 ± 17.6	42.7 ± 21.5	0.291
Adverse events rate, n (%)								
Bleeding	14 (14.3)	6 (8.6)	8 (28.6)	0.021	5 (11.9)	0 (0)	5 (23.8)	0.048
Frequent filter clotting	17 (17.3)	0 (0)	17 (60.7)	< 0.001	12 (28.6)	0 (0)	12 (57.1)	< 0.001
Metabolic acidosis	14 (14.3)	9 (12.9)	5 (17.9)	0.534	5 (11.9)	2 (9.5)	3 (14.3)	1
Metabolic alkalosis	13 (13.3)	7 (10)	6 (21.4)	0.186	7 (16.7)	2 (9.5)	5 (23.8)	0.41
Hypocalcemia	5 (5.1)	2 (2.9)	3 (10.7)	0.139	2 (4.8)	1 (4.8)	1 (4.8)	1
Citrate accumulation	6 (6.1)	6 (8.6)	0 (0)	0.178	1 (2.4)	1 (4.8)	0 (0)	1

CRRT: continuous renal replacement therapy; NA: no anticoagulation; PSM: propensity score matching; RCA: regional citrate anticoagulation.

### Comparative laboratory data of RCA vs. NA

In original cohort, both groups could significantly reduce serum creatinine, serum urea, and platelet (PLT) count after CRRT compared to before CRRT (all P < 0.05), while significantly increasing total calcium level and prolonging aPTT (all P < 0.05). RCA group could also significantly affect serum ALB and serum total bilirubin level after CRRT (all P < 0.05). Hb and prothrombin time (PT) did not change significantly after CRRT in either group (both P > 0.05). No significant differences in pre-/post-treatment changes were observed (all P > 0.05) (Table 3). No significant differences were observed in laboratory indexes between the two groups after matching (Table 4).

**Table 3:** Comparison of Laboratory Indicators Before and After CRRT (Original Cohort)

Group	Time	Total calcium (mmol/L)	Serum creatinine (µmol/L)	Serum urea (mmol/L)	Hb (g/L)	PLT count (× 10^9^/L)	PT (s)	aPTT (s)	Serum ALB (mmol/L)	Serum total bilirubin (mmol/L)
RCA (n = 70)	Before CRRT	2.3 ± 0.2	147.8 ± 95.7^a^	16.5 ± 11.3	104.8 ± 24.0	144.8 ± 79.1	15.0 ± 3.5	30.5 ± 8.7	34.0 ± 6.1	15.8 ± 13.6
	After CRRT	2.5 ± 0.2^b^	100.1 ± 58.2^b^	12.1 ± 7.1^b^	104.1 ± 21.5	104.6 ± 72.1^b^	15.1 ± 3.2	32.3 ± 5.0^b^	36.8 ± 5.1^b^	18.4 ± 7.5^b^
	Difference	−0.1 ± 0.3	16.5 ± 11.3	4.4 ± 8.2	0.8 ± 15.7	40.2 ± 60.6	0.0 ± 3.7	−1.8 ± 8.4	−2.8 ± 6.4	−2.6 ± 14.0
NA (n = 28)	Before CRRT	2.3 ± 0.2	247.8 ± 165.2	19.8 ± 11.1	112.6 ± 21.7	126.9 ± 83.6	14.8 ± 2.4	30.6 ± 7.1	36.0 ± 5.1	18.6 ± 12.5
	After CRRT	2.4 ± 0.2^b^	166.8 ± 100.5^b^	14.9 ± 6.5^b^	106.9 ± 22.8	84.1 ± 54.1^b^	14.6 ± 2.4	33.3 ± 7.2^b^	36.2 ± 6.1	23.6 ± 14.2^b^
	Difference	−0.2 ± 0.3	81.0 ± 113.5	4.9 ± 7.9	5.7 ± 21.3	42.8± 41.7	0.2 ± 2.9	−2.7 ± 8.0	−0.2 ± 7.6	−5.0 ± 12.9

^a^Compared with the NA group before CRRT, P < 0.05. ^b^Compared with in this group before CRRT, P < 0.05. ALB: albumin; aPTT: activated partial thromboplastin time; CRRT: continuous renal replacement therapy; Hb: hemoglobin; NA: no anticoagulation; PLT: platelet; PT: prothrombin time; RCA: regional citrate anticoagulation.

**Table 4:** Comparison of Laboratory Indicators Before and After CRRT (Matched Cohort)

Group	Time	Total calcium (mmol/L)	Serum creatinine (µmol/L)	Serum urea (mmol/L)	Hb (g/L)	PLT count (× 10^9^/L)	PT (s)	aPTT (s)	Serum ALB (mmol/L)	Serum total bilirubin (mmol/L)
RCA (n = 21)	Before CRRT	2.4 ± 0.3	195.2 ± 113.1	20.5 ± 9.8	112.2 ± 25.6	146.6 ± 71.3	14.8 ± 1.5	31.2 ± 6.0	36.1 ± 7.3	15.8 ± 10.5
	After CRRT	2.5 ± 0.2	122.5 ± 72.4^b^	14.3 ± 6.3^b^	112.1 ± 22.9	118.3 ± 90.2^b^	15.2 ± 2.2	34.8 ± 5.3^b^	37.5 ± 4.7	19.8 ± 7.3^b^
	Difference	−0.1 ± 0.3	72.7 ± 51.1	6.1 ± 7.4	0.1 ± 11.4	28.2 ± 53.0	−0.5 ± 3.0	−3.6 ± 6.8	−1.3 ± 6.2	−3.9 ± 10.7
NA (n = 21)	Before CRRT	2.3 ± 0.2	215.3 ± 151.5	20.1 ± 12.7	110.6 ± 21.4	137.3 ± 90.4	14.4 ± 1.9	29.5 ± 5.9	37.0 ± 5.1	16.9 ± 10.0
	After CRRT	2.5 ± 0.3	134.4 ± 73.6^b^	14.1 ± 6.5^b^	107.6 ± 21.5	88.9 ± 56.6^b^	14.6 ± 2.5	34.0 ± 8.1^b^	37.1 ± 6.2	21.8 ± 10.6^b^
	Difference	−0.2 ± 0.3	80.9 ± 92.7	6.2 ± 6.9	3.0 ± 21.3	48.5 ± 45.2	−0.2 ± 2.5	−4.5 ± 7.4	−0.2 ± 7.7	−4.9 ± 11.9

^b^Compared with in this group before CRRT, P < 0.05. ALB: albumin; aPTT: activated partial thromboplastin time; CRRT: continuous renal replacement therapy; Hb: hemoglobin; NA: no anticoagulation; PLT: platelet; PT: prothrombin time; RCA: regional citrate anticoagulation.

### Analysis of the first filter lifespan and associated risk factors

**Figure 2:**
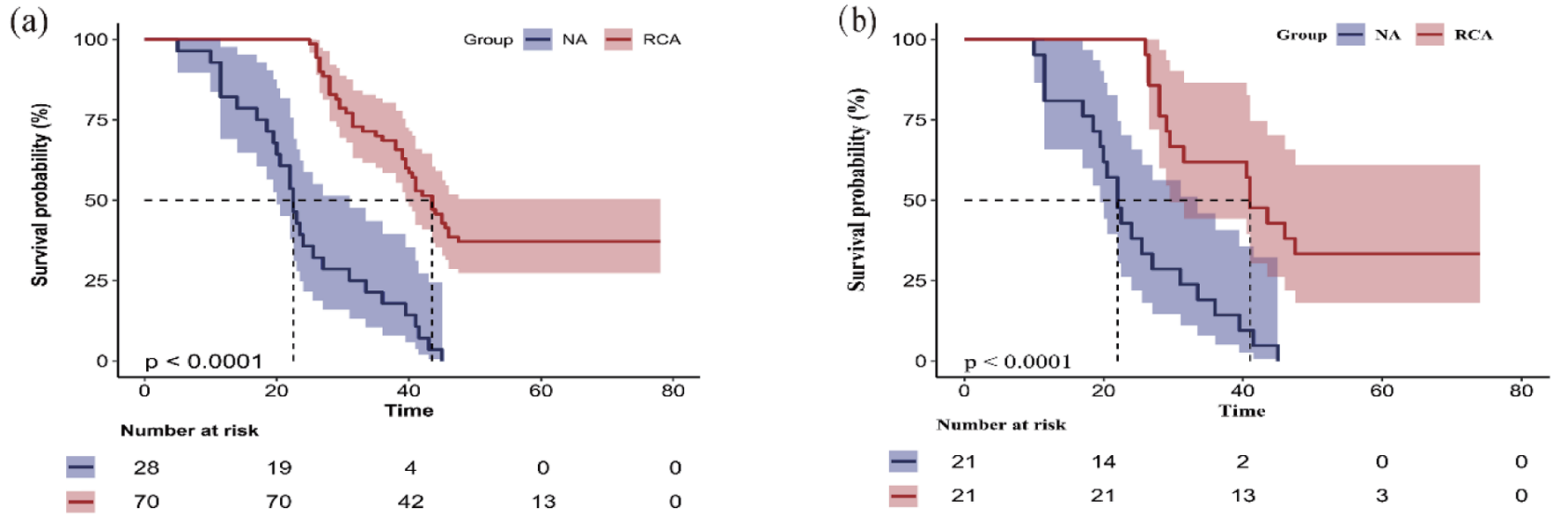
Kaplan-Meier curve shows the filter lifespan of the first filter of the regional citrate anticoagulation (RCA) group and no anticoagulation (NA) group in the original cohort (a) and the matched cohort (b). The Kaplan-Meier survival curves demonstrated significant differences in first filter lifespan between the two anticoagulation groups. The overall median filter survival time was 39.0 h (95% CI, 34.1–43.9). Median filter lifespan was 22.5 h in NA group (95% CI 19.9–25.1). Median lifespan was significantly longer at 43.5 h in RCA group (95% CI, 39.8–47.2) (a). Kaplan-Meier survival curves after matching. The first filter lifespan was considerably longer in the RCA group compared to the NA group (P < 0.001) (b). 95% CI: 95% confidence interval.

The Kaplan-Meier survival curves demonstrated significant differences in first filter lifespan between the two anticoagulation groups. The overall median filter survival time was 39.0 h (95% CI, 34.1–43.9). Median filter lifespan was 22.5 h in NA group (95% CI, 19.9–25.1). Median lifespan was significantly longer at 43.5 h in the RCA group (95% CI, 39.8–47.2) (Fig. 2a). Kaplan-Meier survival curves after matching showed that the first filter lifespan was considerably longer in the RCA group compared to the NA group (P < 0.001) (Fig. 2b).

**Figure 3:**
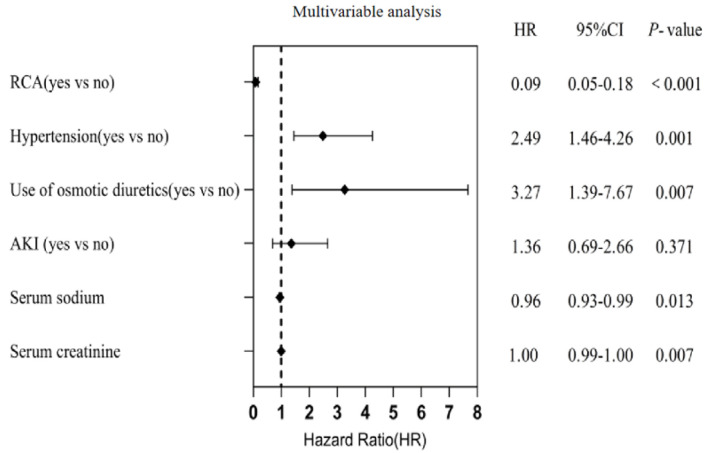
Multivariable Cox proportional risk model regression analysis affecting the lifespan of the first filter. Regional citrate anticoagulation (RCA) remained a significant independent predictor of prolonged first filter lifespan after adjustment for covariates (HR = 0.09, 95% CI, 0.05−0.18). 95% CI: 95% confidence interval; HR: hazard ratio.

As shown in Table 5 and Fig. 3, VIFs for all covariates were < 5, confirming the absence of significant multicollinearity in the multivariable Cox regression analysis. RCA remained a significant independent predictor of prolonged first filter lifespan after adjustment for covariates (HR = 0.09, 95% CI, 0.05–0.18).

**Table 5:** Univariate and Multivariable Cox Regression Analyses of the Risk Factors of the First Filter Lifespan (Original Cohort)

Variables	Univariate analysis			Multivariable analysis		
	HR	95% CI	P value	HR	95% CI	P value
RCA (yes vs. no)	0.19	0.11–0.31	< 0.001	0.09	0.05–0.18	< 0.001
Hypertension (yes vs. no)	2.08	1.29–3.35	0.003	2.49	1.46–4.26	0.001
Use of osmotic diuretics (yes vs. no)	2.19	1.00–4.78	0.05	3.27	1.39–7.67	0.007
AKI (yes vs. no)	2.22	1.23–3.98	0.008	1.36	0.69–2.66	0.371
Serum sodium	0.96	0.93–0.99	0.006	0.96	0.93–0.99	0.013
Serum creatinine	1.002	1.0005–1.0039	0.012	1.00	0.99–1.00	0.007

Note: The multivariable Cox proportional hazards model was constructed by including all variables with a P-value < 0.05 in the univariate analysis (i.e., RCA, hypertension, use of osmotic diuretics, AKI, serum sodium, and serum creatinine). The variance inflation factor (VIF) for each covariate in the final model was < 5, indicating no significant multicollinearity. AKI: acute kidney injury; CI: confidence interval; HR: hazard ratio; RCA: regional citrate anticoagulation.

### Comparative hospitalization

In the original cohort, the RCA group had longer ICU stay days than the NA group, but there was no significant difference (21.0 ± 17.5 vs. 15.7 ± 11.7, P = 0.146). However, the RCA group had significantly higher hospitalization costs (258,761.0 ± 240,991.8 vs. 157,816.6 ± 89,807.1, P = 0.034). In-hospital mortality did not differ significantly between the RCA and NA groups (14.3% vs. 28.6%, P = 0.099). Similarly, there was no significant difference in 28-day mortality between the two groups (60% vs. 75%, P = 0.162) (Table 6). After PSM, no significant differences were observed in any hospitalization-related outcomes between the two groups.

**Table 6:** Comparison of Hospitalization Before and After PSM

Variables	Before PSM				After PSM			
	Total (n = 98)	RCA (n = 70)	NA (n = 28)	P value	Total (n = 42)	RCA (n = 21)	NA (n = 21)	P value
Days in ICU	19.5 ± 16.1	21.0 ± 17.5	15.7 ± 11.7	0.146	15.9 ± 10.6	16.4 ± 11.5	15.3 ± 10.0	0.732
Costs in hospital, yuan	229,919.7 ± 213,678.5	258,761.0 ± 240,991.8	157,816.6 ± 89,807.1	0.034	192,082.6 ± 138,796.6	228,431.1 ± 168,930.5	155,734.1 ± 90,444.3	0.09
In-hospital mortality	18 (18.4)	10 (14.3)	8 (28.6)	0.099	9 (21.4)	2 (9.5)	7 (33.3)	0.13
Mortality on 28 days	63 (64.3)	42 (60)	21 (75)	0.162	31 (73.8)	16 (76.2)	15 (71.4)	0.726

ICU: intensive care unit; NA: no anticoagulation; PSM: propensity score matching; RCA: regional citrate anticoagulation.

## Discussion

In neurocritically ill patients with chronic severe hypernatremia and high bleeding risk, the selection of anticoagulation for CRRT remains debated. This study demonstrated that RCA achieved a comparable sodium-lowering effect to the NA group, while exhibiting a reduced risk of bleeding and filter clotting. Paradoxically, the incidence of bleeding events was significantly higher in the NA group than in the RCA group, which may be explained by the consequences of frequent circuit clotting. Each clotting event led to circuit replacement and inherent blood loss, which, when recurrent, resulted in cumulative iatrogenic blood loss and could manifest as a bleeding event. Notably, no significant citrate accumulation was observed, supporting the safety and efficacy of RCA in this population. Although hypernatremia is traditionally regarded as a relative contraindication to RCA [12], concerns primarily stem from the high sodium load of 4% TSC solution (408 mmol/L sodium). Despite this theoretical risk, our findings revealed that the serum sodium correction rate in the RCA group aligned with the NA group, both adhering to the recommended targets. Thus, CRRT effectively regulates serum sodium levels regardless of anticoagulation strategy, and RCA does not exacerbate hypernatremia.

Unplanned circuit clotting, primarily resulting from extracorporeal circuit coagulation, is the most frequent complication during CRRT. This leads to reduced treatment duration, compromised efficacy, and failure to achieve therapeutic goals [13]. Randomized controlled trials have consistently shown that citrate anticoagulation effectively prolongs filter lifespan while reducing complication rates, treatment costs, and interruptions [14]. Neurocritical patients—particularly those with trauma, postoperative neurosurgical status, active bleeding, or coagulopathy—face heightened bleeding risks, precluding systemic anticoagulation. In this study, the RCA group demonstrated significantly longer CRRT duration and filter survival compared to the NA group, ensuring the attainment of treatment goals. By mitigating unplanned clotting, RCA minimizes interruptions and enhances procedural continuity [15]. Notably, the NA group exhibited post-CRRT declines in Hb and PLT levels, although these changes did not reach statistical significance.

RCA may trigger metabolic abnormalities, including metabolic alkalosis, metabolic acidosis, and electrolyte disturbances. These abnormalities stem from factors such as citrate overload, inadequate citrate metabolism, citrate loss in waste fluid, and calcium chelation by citrate [16]. Moreover, the application of RCA is associated with the risk of citrate accumulation [17]. Implementing standardized protocols for adjusting dialysate flow rates and supplementing calcium can effectively keep blood pH and intracellular ionized calcium (iCa^2+^) levels within the normal range, thereby reducing the occurrence of metabolic complications [18]. In this study, blood gas analysis was carried out every 4 h, and prescription parameters were adjusted immediately according to the results. The incidence of hypocalcemia showed no significant difference between the two groups, and no patients exhibited clinical symptoms of citrate accumulation.

In this study, the mortality rates in the RCA group were persistently lower than those in the NA group. However, the differences in in-hospital and 28-day mortality rates between the two groups did not reach statistical significance. The application of RCA enhanced the short-term prognosis by decreasing interruptions in CRRT caused by recurrent extracorporeal circuit coagulation. Nevertheless, patients in the RCA group experienced longer ICU stays and higher hospitalization costs [19]. It is crucial to note that these findings should not be interpreted as a negative effect of RCA. Instead, they may be influenced by residual confounding, as the significantly longer CRRT duration achieved in the RCA group inherently contributes to higher resource utilization. This longer treatment time reflects the success of RCA in maintaining continuous life-sustaining therapy, thereby allowing patients to remain in the ICU and accrue associated costs. In contrast, the high rate of treatment failure in the NA group could have led to earlier cessation of CRRT. Therefore, these parameters are influenced by multiple factors and do not serve as valid measures of the anticoagulation strategy’s effect on patient prognosis. When compared with the results from other centers, the mortality rates in this study were generally higher. This disparity can likely be attributed to two main factors. Firstly, the neurocritical patients included in this study had a relatively higher bleeding risk. Secondly, a significant number of patients’ families opted to withdraw from treatment because of the substantial financial burden.

Compared with previous studies, our research presents several distinct advantages. Firstly, to mitigate the influence of imbalanced baseline characteristics on the study results, we specifically selected neurocritically ill patients with a heightened bleeding risk. This targeted patient inclusion is a key strength of our research. Secondly, prior investigations have mainly concentrated on assessing the efficacy and safety of RCA therapy during CRRT in patients with liver failure [20, 21] and severe lactic acidosis [22, 23]. Research has indicated that the incidence of severe hypernatremia is notably lower than that of liver failure and severe lactic acidosis. Only a limited number of studies reported on CRRT use in patients with acute severe hypernatremia and evaluated the effectiveness of different anticoagulation strategies [19]. Moreover, even fewer studies have focused on chronic severe hypernatremia. Consequently, our study offers valuable insights for clinicians regarding the application of CRRT in patients with chronic severe hypernatremia and heightened bleeding risk.

The retrospective design of this study is its most significant drawback. Retrospective cohort studies often face the issue of incomplete laboratory data. Furthermore, due to the retrospective nature of this study and the specific challenges in a neurocritical care population, data on certain baseline characteristics such as body mass index (BMI), smoking status, and alcohol history were not systematically available and could not be included in our analysis. Serum phosphate levels were closely monitored during treatment. When serum phosphate fell below 1.45 mmol/L, supplementation was provided by adding 1.25 mL of compound potassium phosphate (containing 4.0 mmol of phosphorus) to the replacement fluid. This adjustment maintains the phosphorus concentration in the replacement fluid at approximately 1.0 mmol/L [24]. While these factors could potentially influence outcomes, the successful balancing of all other available major clinical and laboratory covariates through PSM strengthens the internal validity of our primary findings regarding filter lifespan and bleeding events. Another limitation lies in the study’s small sample size and single-center nature. A small-scale study may not be representative enough to draw broad and generalizable conclusions. With a limited number of participants, the results may be more likely to be affected by random fluctuations. Moreover, being a single-center study, it may have unique patient populations, treatment protocols, and resource availabilities that are not applicable to other medical settings. The selection of anticoagulation method was based to the attending physicians’ preferences and the patient’s financial situation. This subjective decision-making process can lead to both systemic and selection biases. Specifically, systemic bias may occur because different physicians may have different understandings and practices regarding anticoagulation, which can influence the overall treatment outcomes in a non-random way; meanwhile, selection bias can arise as physicians may choose different anticoagulation methods based on their preconceived notions about patient characteristics, potentially leading to an uneven distribution of patients in different treatment groups. This could further compromise the validity and reliability of the study results.

### Conclusion

RCA is safer and equally effective as NA for CRRT in neurocritical patients with chronic severe hypernatremia, reducing bleeding and filter clotting risks. While our retrospective study suggests that RCA is a safe and effective strategy in this population, the findings require validation in a large-scale, randomized controlled trial to establish conclusive evidence.

## Data Availability

The dataset supporting the conclusions of this article is included within the article and its additional file.
